# Generalized Growth of Estuarine, Household and Clinical Isolates of *Pseudomonas aeruginosa*

**DOI:** 10.3389/fmicb.2018.00305

**Published:** 2018-02-27

**Authors:** Kelly E. Diaz, Susanna K. Remold, Ogochukwu Onyiri, Maura Bozeman, Peter A. Raymond, Paul E. Turner

**Affiliations:** ^1^Department of Ecology & Evolutionary Biology, Yale University, New Haven, CT, United States; ^2^Department of Biology, University of Louisville, Louisville, KY, United States; ^3^Yale School of Forestry & Environmental Studies, Yale University, New Haven, CT, United States; ^4^Program in Microbiology, Yale School of Medicine, New Haven, CT, United States

**Keywords:** bacteria, evolutionary ecology, fitness, genotype by environment interaction, opportunistic pathogen

## Abstract

*Pseudomonas aeruginosa* is an opportunistic pathogen of particular concern to immune-compromised people, such as cystic fibrosis patients and burn victims. These bacteria grow in built environments including hospitals and households, and in natural environments such as rivers and estuaries. However, there is conflicting evidence whether recent environments like the human lung and open ocean affect *P. aeruginosa* growth performance in alternate environments. We hypothesized that bacteria recently isolated from dissimilar habitats should grow differently in media containing artificial versus natural resources. To test this idea, we examined growth of *P. aeruginosa* isolates from three environments (estuary, household, and clinic) in three media types: minimal-glucose lab medium, and media prepared from sugar maple leaves or big bluestem grass. We used automated spectrophotometry to measure high-resolution growth curves for all isolate by media combinations, and studied two fitness parameters: growth rate and maximum population density. Results showed high variability in growth rate among isolates, both overall and in its dependence on assay media, but this variability was not associated with habitat of isolation. In contrast, total growth (change in absorbance over the experiment) differed overall among habitats of isolation, and there were media-specific differences in mean total growth among habitats of isolation, and in among-habitat variability in the media-specific response. This was driven primarily by greater total growth of estuary isolates when compared with those from other habitats of origin, and greater media-specific variability among household isolates than those from other habitats of origin. Taken together, these results suggest that for growth rate *P. aeruginosa* bacteria appear to be broad generalists without regard to current or recent habitat, whereas for total growth a signature of recent ecological history can be detected.

## Introduction

*Pseudomonas aeruginosa* bacteria thrive in a wide variety of environments, and can be free-living in both natural and built-environment settings (Kimata et al., 2003; Khan et al., 2007; Remold et al., 2011; Kidd et al., 2012; Selezska et al., 2012; Purdy-Gibson et al., 2015). In addition, these bacteria are opportunistic pathogens of humans, especially important in respiratory and urinary tract infections of immune-compromised individuals, such as premature infants, elderly persons, and chemotherapy patients (Høiby, 2006; Kirov et al., 2007; Mesaros et al., 2007; Kokare et al., 2009; Schechner et al., 2009). *P. aeruginosa* can be difficult to treat if multi-drug resistant (Chan et al., 2016), and can cause high mortality in infected cystic fibrosis (CF) patients (Bazire et al., 2007) due to dense bacterial growth that obstructs lung airways and also prevents efficient phagocytosis of the bacteria by the immune system. For patients with CF, there are many instances in which infecting strains are genetically more similar to environmental isolates than to strains from other chronically infected individuals (Burns et al., 2001; Jelsbak et al., 2007; Workentine and Surette, 2011; Rau et al., 2012), indicating that the environment is an important potential source of infecting strains. Although *P. aeruginosa* is found in myriad environments (Yoshpe-Purer and Golderman, 1987; Remold et al., 2011; Purdy-Gibson et al., 2015), it is unclear whether isolates tend to grow efficiently in their current environment, but poorly elsewhere. That is, does recent ecological history affect *P. aeruginosa* growth performance across habitats?

*Pseudomonas aeruginosa* bacteria that are recently isolated from different environments may or may not show performance differences when grown under a common environmental challenge. On the one hand, local adaptation of *P. aeruginosa* may produce adaptive traits that are useful for the selective environment and neutral or deleterious elsewhere. This scenario assumes that *P. aeruginosa* bacteria tend to suffer growth trade-offs across environments. For example, *P. aeruginosa* isolated from the lungs of CF patients often show changes in a variety of traits, including motility, bacteriocin use, sensitivity to phagocytosis by macrophages and predation by protozoa, and susceptibility to infection by various bacteriophages (Mahenthiralingam et al., 1994; D’Argenio et al., 2007; Ciofu et al., 2010; Friman et al., 2013; Ghoul et al., 2015; France and Remold, 2015); although largely untested, these altered traits may affect bacterial fitness in other habitats. Similarly, *P. aeruginosa* isolates taken from the ocean show Na^+^ pump changes that would seem only to foster better survival under high salinity and low nutrient conditions typical of most marine ecosystems (Kimata et al., 2003). On the other hand, *P. aeruginosa* isolates may possess traits that are neutral or beneficial (on average) in other locales, causing their performance across environments to be uncorrelated with their habitat of isolation (Ruimy et al., 2001). In addition, substantial variability among isolates obtained from a single environment has been documented (e.g., Workentine and Surette, 2011; Kidd et al., 2012), with the possibility that different types of environments support different amounts of phenotypic diversity among the *P. aeruginosa* bacteria present. *P. aeruginosa* is undoubtedly a ‘generalist’ bacterium that can thrive in environments as different as an estuary and the human lung. However, this may be explained by widespread prevalence either of ecotypes (isolates adapted to their specific environment, and maladapted elsewhere), or of generalist *P. aeruginosa* bacteria that grow well across environments.

To determine which explanation best describes the biology of *P. aeruginosa*, we used a collection of 11 isolates drawn from clinical, household, and estuary environments, and compared their performance (growth rate, maximum growth) in media prepared from two natural plant sources, as well as in a defined minimal-glucose lab medium. It has been long known that *P. aeruginosa* bacteria vary in their ability to use different resources (Schmidt et al., 1996; Frimmersdorf et al., 2010). But the main goal and novelty of the current study were to compare growth of bacterial isolates drawn from different environments in plant-derived media. In particular, the naturally derived, undefined media used in this study were chosen because leaves and grasses often provide the dissolved organic matter for bacteria growing in freshwater and estuarine aquatic systems (Qualls, 2000).

## Materials and Methods

### Bacteria and Culture Conditions

*Pseudomonas aeruginosa* bacteria used in this study are listed in **Table 1**. The four estuary isolates (#E1-E4; kindly provided by M. Polz, Massachusetts Institute of Technology) were obtained in 2006 from Plum Island Estuary, MA, United States. The four household isolates (#H1-H4) were collected in 2005-07 from kitchen sink drains of four different houses in Louisville, KY (Purdy-Gibson et al., 2015). Two clinical isolates (#C2-C3; kindly provided by B. Kazmierczak, Yale School of Medicine) were collected in 2010 from patient wounds and lungs (Murray et al., 2010). All of the aforementioned isolates were stored at -80°C immediately after isolation and the current study employed samples from frozen stock; this design was to minimize any confounding effects of isolate adaptation to lab conditions. The remaining clinical isolate was PA01 (#C1; a.k.a. #ATCC-47085, American Type Culture Collection, Manassas, VA, United States), a popular laboratory model for examining biology of *P. aeruginosa*, which was originally designated ‘strain 1’ and taken from a patient wound isolated in Melbourne, Australia (Holloway, 1955; Klockgether et al., 2010). Although #C1 was derived from the popular lab model PA01, it was a low-passage isolate that was not previously subjected to prolonged serial transfer in the lab; nevertheless, some generalized adaptation to lab conditions could not be ruled out for #C1. For all 11 isolates, the *P. aeruginosa* species designation was confirmed via 16S rRNA sequencing; additionally, the four estuary isolates were confirmed for growth ability on *Pseudomonas* isolation agar (Sigma–Aldrich, St. Louis, MO, United States), and two of the clinical isolates (#C2-C3) were confirmed by clinical microbiology laboratory assays (data not shown; B. Kazmierczak personal communication).

**Table 1:** Bacteria designations by isolation environment.

Isolate name	Original isolate designation^1^	Source of isolation
**Estuary isolates**		
E1	D02	Plum Island Estuary, MA, United States
E2	A11	Plum Island Estuary, MA, United States
E3	B12	Plum Island Estuary, MA, United States
E4	C02	Plum Island Estuary, MA, United States
**Household isolates**		
H1	SRP1175	Purdy-Gibson et al., 2015
H2	SRP1258	Purdy-Gibson et al., 2015
H3	SRP1435	Purdy-Gibson et al., 2015
H4	SRP1607	Purdy-Gibson et al., 2015
**Clinical isolates**		
C1	PA01 (ATCC #47085)	Holloway, 1955
C2	AP1236	Murray et al., 2010
C3	AP1251	Murray et al., 2010

*^*1*^The original designations for estuary isolates correspond to those given by the Polz Lab at the Massachusetts Institute of Technology in Cambridge, MA, United States. Only isolate C1 is currently deposited in a strain collection: American Type Culture Collection (ATCC).*

Bacteria were grown from frozen stock on 1.5% agar made from Luria-Bertani broth (10 g Bacto^TM^ tryptone, 5 g Bacto^TM^ yeast extract, and 10 g NaCl L^-1^), by streaking for single colonies that were grown for 24 h at 37°C. Cultures were prepared by looping a colony into 10 mL of Davis-Minimal medium (7 g potassium phosphate dibasic trihydrate, 2 g potassium phosphate monobasic anhydrous, 1 g ammonium sulfate, 1 g sodium citrate, 1 mL 10% [10 g/100 mL DI water] magnesium sulfate, and 1 mL 0.2% [0.2g/100 mL DI water] thiamine hydrochloride L^-1^), with 250 μL of 10% (10 g/100 mL DI water) glucose L^-1^ as the only added sugar. Cultures were grown overnight with shaking (150 rpm) and 37°C incubation. All media reagents were purchased from ThermoFisher Scientific (Waltham, MA, United States).

### Challenge Media

Sugar maple (*Acer saccharum*) leaves were picked when senescent from a single tree located on Yale University campus (New Haven, CT, United States). Big bluestem grass (*Andropogon gerardii*) was grown at Yale University, collected and air-dried. To prepare sugar maple and big bluestem leachates, ∼38 g plant material were soaked in 1 L Milli-Q (Merck Millipore, Burlington, MA, United States) water for ∼24 h at 4°C. Plant material was removed, and leachates were filtered sequentially through 0.7 μm GF/F, followed by polycarbonate membrane filter (0.2 μm) that were pre-soaked and thoroughly rinsed with Milli-Q water. Using estimates (see Results) for sugar maple leachate and big bluestem leachate concentrations of 203.3 mg C L^-1^ and 59 mg C L^-1^, each leachate was diluted to ∼25 mg dissolved organic carbon (DOC) L^-1^ (i.e., ∼25 ppm). Working stocks (1 L) were created by mixing diluted leachates with a combination of inorganic nutrients (“COMBO”) commonly used in ecological experiments (Kilham et al., 1998): CaCl_2_ 2 H_2_O (36.76 mg L^-1^), MgSO_4_ 7 H_2_O (36.97 mg L^-1^), K_2_HPO_4_ (8.71 mg L^-1^), NaNO_3_ (85.01 mg L^-1^), NaHCO_3_ (12.60 mg L^-1^), Na_2_SiO_3_ 9 H_2_O (28.42 mg L^-1^), H_3_BO_3_ (24.00 mg L^-1^), and KCl (7.45 mg L^-1^). Leachates were refrigerated and used within 5 days after preparation. The minimal-glucose lab medium was Davis Minimal medium following the above recipe with 250 μL of 10% glucose (10 g/100 mL DI water) L^-1^ as the only added sugar (abbreviated as DM10).

### Growth Curve Analysis

Growth curves were obtained using an automated spectrophotometer (microplate reader model ES2000; Tecan Group Ltd., Mannedorf, Switzerland). To measure growth, 4 μL (∼10^6^ colony-forming units) of a test isolate was mixed with 196 μL of assay medium in a single well of a flat-bottomed 96-well plate (Corning Inc., Corning, NY, United States). Each of the 11 bacterial isolates was assayed four times on the plate, and the plate contained four control wells consisting of 200 μL bacteria-free assay medium. Optical density (600 nm wavelength; OD_600_) of each well was measured every 10 min for 137 cycles at 37°C, with shaking (60.6 rpm, 2.5 mm amplitude) imposed in between measures. After 24 h, high-resolution growth curves were obtained. All curves were inspected by eye, and some replicate growth curves were excluded because their OD_600_ values fluctuated erratically over time, indicating that air bubbles confounded automated measures of optical densities. Two summary parameters (total growth; maximum growth rate) were extracted from each curve. Total growth was calculated as the difference between the maximum and initial OD_600_. Maximum growth rate was fitted with the exponential model using the Curve Fitter program^1^ (Delaney et al., unpublished), using OD_600_ readings falling between the minimum OD_600_ and 75% of the maximum absorbance values.

### Statistical Analysis

In two sets of analyses, we used mixed linear models (SAS Institute Inc., 2011) to test the dependence of our two response variables, maximum growth rate, and total growth achieved (the latter natural log transformed to improve normality), on the fixed factors habitat of origin, assay media and their interaction, and the random factors isolate nested within habitat of origin, and the isolate by assay media type interaction nested within habitat of origin. In these models, the nested random factor was fit with and without the assumption of equal variances among levels of the habitat of origin, to test contributions of differences in variance among isolates within habitats of origin, and differences in means among habitats of origin on the variability in the response variables. Significant tests for fixed effects were explored using linear contrasts employing a Tukey–Kramer correction for multiple comparisons.

## Results

### Dissolved Organic Carbon in Leachate Media

We conducted preliminary assays to measure the average dissolved organic carbon (DOC) contained in leachates prepared from leaves of sugar maple (*Acer saccharum*) and big bluestem grass (*Andropogon gerardii*), two native North American plants that serve as possible sources of nutrient run-off entering rivers and estuaries. DOC in sugar maple leachate was 203.3 mg/L, and that of big bluestem leachate was 59.0 mg/L. Also, DOC in the COMBO medium alone was observed to be 0.4 mg/L. We concluded that the sugar maple leachate had a relatively higher DOC content (see also Bozeman, 2012).

### Growth Parameters

The high-resolution growth curves measured on the automated spectrophotometer were analyzed to compare and contrast the growth performance of the 11 bacterial isolates in each of the three media types. Because growth performance can be interpreted in multiple ways, comparisons between the isolates were made using two summary parameters that address different aspects of performance: the maximum growth rate of each isolate in each medium was assessed using Curve Fitter^2^, and the total growth was measured as total change in absorbance in each medium type. The latter is a measure of population density the isolate can achieve in a particular medium.

**FIGURE 1:**
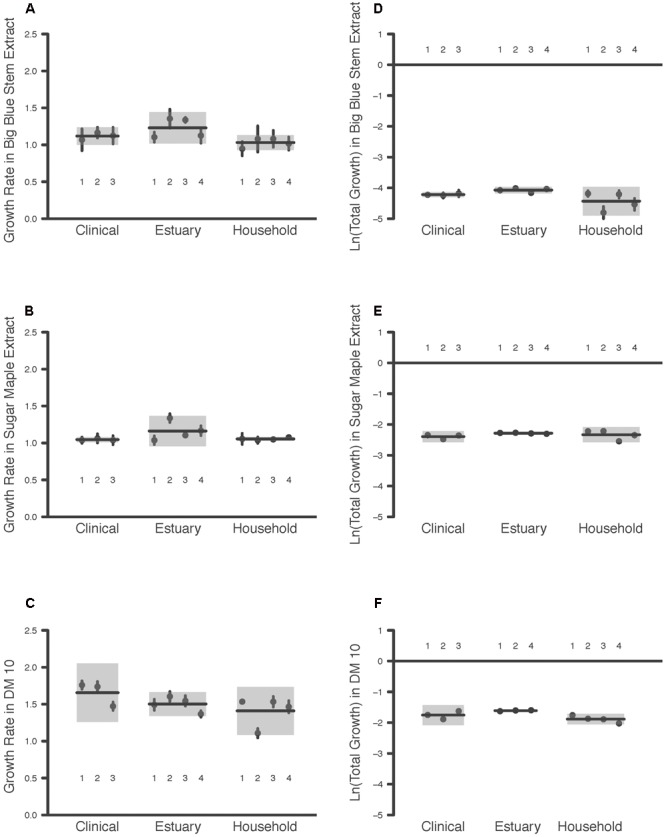
Maximum Growth Rate and Total Growth of 11 *P. aeruginosa* Isolates in Three Media Types. Each point is the raw mean and 95% C.I. of four replicate measures per *P. aeruginosa* isolate, for bacteria taken from clinical, estuary and household environments. Two components of bacterial growth, the maximum growth rate and the total growth, were estimated from growth curve measurements of bacterial populations between 0 and 24 h (see text for details). Isolates are numbered for comparison among panels. Each line indicates the group mean, and each gray box indicates the 95% C.I. of the strains from a given environment. Maximum growth rates of bacteria were estimated in media prepared from **(A)** big bluestem grass, **(B)** sugar maple leaves, and **(C)** 10 μg/mL glucose. Total growth of bacteria was estimated in media prepared from **(D)** big bluestem grass, **(E)** sugar maple leaves, and **(F)** 10 μg/mL glucose.

The maximum growth rate achieved by the *P. aeruginosa* studied was highest overall in DM10, followed by big bluestem leachate and then by sugar maple leachate (Media effect, **Table 2A**, all comparisons significant after Tukey–Kramer correction; **Figures 1A–C**). There were no significant differences in growth rate associated with habitat of origin (Origin effect, **Table 2B**), and although there was a marginally significant Origin^∗^Media effect (**Table 2B**), contrasts within this effect detected no habitat of origin-specific differences in responses to the same media. Furthermore, there were no differences among habitats of origin for isolate variability in growth rate, either overall or in specific media (Isolate (Origin) *[variance]* and Isolate^∗^Media(Origin) *[variance]* effects, **Table 2A**). This absence of mean differences and differences in variability among habitats of origin does not reflect low overall variation in growth rate; in fact, there were highly significant differences among isolates within habitats of origin in their overall and media-specific growth (Isolate(Origin) *[mean]* and Isolate^∗^Media(Origin) *[mean]* effects, **Table 2A**). Overall, these results were consistent with the hypothesis that *P. aeruginosa* isolates were highly variable in growth and that no particular association existed between growth ability and habitat of origin; therefore, our results for maximal growth rate did not suggest that the prior environment of isolates affected their growth performance.

**Table 2:** Dependence of *P. aeruginosa* populations’ growth rate **(A)** and ln-transformed total growth achieved **(B)** on the fixed factors habitat of origin, assay media and their interaction, and the random factors isolate and the interaction of isolate with assay media, as determined using mixed linear models.

Source	DF^§^	Test statistic^‡^
**(A) Growth rate**		
Origin	2, 5.7	3.0^NS^
Media	2,16	61.6^∗∗∗^
Origin^∗^Media	4,16	2.6^+^
Isolate (Origin) *[means]*	1	32.3^∗∗∗^
Isolate (Origin) *[variances]*	2	0.6^NS^
Isolate^∗^Media (Origin) *[means]*	1	98.4^∗∗∗^
Isolate^∗^Media (Origin) *[variances]*	2	1.5^NS^
**(B) Ln(Total Growth)**		
Origin	2, 4.5	18.89^∗∗^
Media	2, 11.5	1302.4^∗∗∗^
Origin^∗^Media	4, 10.1	1.2^∗∗∗^
Isolate (Origin) *[means]*	1	20.0^∗∗∗^
Isolate (Origin) *[variances]*	2	1.4^NS^
Isolate^∗^Media (Origin) *[means]*	1	130.3^∗∗∗^
Isolate^∗^Media (Origin) *[variances]*	2	17.6^∗∗∗^

*^§^DF indicates degrees of freedom, denominator DF for *F*-test is estimated using the Satterthwaite approximation. DF for LR tests are equal to the difference in the number of parameters in the full and reducted models. ^‡^The fixed effect is tested with an approximate *F* test. Population (history) *[variances]* is tested using likelihood ratio (LR) tests; the LR test statistic is -2 × (maximum likelihood of the test’s full model – maximum likelihood of the restricted model, from which the variance component being tested has been removed), and is distributed approximately chi-squared. In the tests of variance effects, variances are constrained to be equal in the reduced model. Key for reported statistical-significance values: ^*NS*^*p* > 0.1; ^+^, 0.05 < *p* < 0.1; ^∗^, 0.01 < *p* < 0.05; ^∗∗^, 0.001 < *p* < 0.01; ^∗∗∗^, *p* < 0.001.*

Consistent with the substantial differences in DOC they contain, the total growth detected in the three growth media differed significantly overall (Media effect, **Table 2B**). Pairwise contrasts correcting for multiple comparisons indicated that sugar maple leachate supported more growth than big bluestem leachate, followed by DM10 (**Figures 1D–F**, all comparisons significant after Tukey–Kramer correction for multiple comparisons). Isolates from the estuary achieved significantly higher total growth than household isolates, and marginally significantly higher growth than clinical isolates (Origin effect, **Table 2B**). Overall differences in *P. aeruginosa* growth among media types depended on the habitat of origin (Origin^∗^Media effect, **Table 2B**); this was driven in part by poorer growth of household *P. aeruginosa* on big bluestem leachate than on the other two media (**Figures 1D–F**, *p* < 0.001 for both contrasts after correction for multiple comparisons), and by marginally significantly higher growth of estuary isolates than household isolates on big bluestem leachate (*p* = 0.08 after correction for multiple comparisons). There were also significant differences among isolates within habitats of origin in their overall and media-specific growth (Isolate(Origin) *[mean]* and Isolate^∗^Media(Origin) *[mean]* effects, **Table 2B**), consistent with other studies showing metabolic diversity among *P. aeruginosa* (Palmer et al., 2010; Rodríguez-Rojas et al., 2012; Jørgensen et al., 2015). However, the model also indicated that habitats of origin differed in isolate to isolate variability in the dependence of growth on media type (Isolate^∗^Media(Origin) *[variance]* effect, **Table 2B**), with household isolates being substantially more variable in their media-specific response, and isolates from the estuary and the clinical isolates being more consistent with others from their habitat of origin.

Last, we generated Supplementary Figure S1 that depicts a scatterplot of the two growth parameter estimates addressed in the study. The results presented in **Figure 1** and **Table 2** are clearly seen in Supplementary Figure S1 as well. First, there is a stronger separation by Media than by Habitat of Origin both vertically (total growth) and horizontally (growth rate). Second, there is some clustering of isolates from the same habitat of origin within the larger clusters of assay Media (indicating a Media^∗^Origin interaction effect), also seen along both axes.

## Discussion

There is accumulating evidence for extensive phenotypic variability among *P. aeruginosa* bacteria isolated from various types of environments. Here we address two competing hypotheses: (i) that *P. aeruginosa* bacteria are broad generalists, and that the phenotypic variability among isolates is not associated with current or recent ecology, and (ii) that isolates from the same habitat are more phenotypically similar to one another than they are to isolates from different habitats. Related to this second question, we hypothesized that the habitat types may also differ in the extent of phenotypic variability among their associated isolates.

We evaluated these hypotheses by comparing two growth parameters, maximum growth rate and total growth achieved, of 11 *P. aeruginosa* isolates from three habitats of isolation (human household, estuary and clinical), when grown in three assay media. These media types included a commonly used laboratory medium with glucose as the input resource (DM10), and two undefined media chosen for their ecological relevance: leachate of sugar maple leaves (*Acer saccharum*), and leachate of big bluestem grass (*Andropogon gerardii*). These two perennial plant species are commonly found in North America, including in estuary environments; sugar maple occurs in the United States and Canada in Central North America to the Atlantic coast, while big bluestem grass has a broader distribution that includes the United States and Canada east of the Rocky Mountains.

We observed that results for the two growth parameters differed in terms of the hypothesis that was best supported. Maximal growth rate was highly variable overall, without any association with habitat of origin; these data best-supported hypothesis (i). In contrast, total growth showed differences among habitats of origin in overall performance, media-specific performance, and media-specific among-isolate variability. Thus, the results for total growth best-supported hypothesis (ii).

Regarding variability in total growth among habitats of isolation, there are patterns that are consistent with known habitat differences. For example, the higher overall total growth achieved by estuary isolates may reflect selection for greater efficiency in resource-use driven by living in an environment with dynamic resource levels; DOC composition tends to vary along estuarine salinity gradients due to factors such as mixing of water masses from freshwater and saltwater sources (e.g., Raymond and Hopkinson, 2003). Therefore, the marginally significantly better performance of these isolates compared to household isolates on big bluestem leachate suggests the influence of the habitat of origin. A second example is the greater variability among household isolates in media-specific total growth, which may reflect varying conditions among the household environments from which these bacteria were isolated, compared to those taken from estuary and clinical settings. These ideas are highly speculative, however; a thorough analysis of resource quality and variability among isolation habitats extends beyond the scope of the current study, and it is difficult to draw strong conclusions given the small sample sizes of isolates taken from each habitat.

Nevertheless, it is important to recognize that higher growth rate may provide a relative fitness advantage to bacteria in one environment, while higher total growth may be more advantageous in another environment, because each parameter conveys a unique fitness advantage (Buchanan et al., 1997). This idea relates to the general evolutionary theory prediction that selection for growth rate should be influenced by the presence/absence of competitors; strong competition should promote evolution of higher growth rate to outpace competitors, which may trade off against overall growth yield (see Mueller et al., 1991 and Novak et al., 2006 for empirical examples). Thus, the degree to which local adaptation can evolve for these two bacterial traits may be quite different, depending on the extent of local competition or other factors. For example, the evolution of higher maximal growth rate suggests an increased metabolic demand, which may trade-off with the ability to divert metabolism to overcoming stress tolerance, prolonging survival during starvation, and meeting other challenges. These myriad challenges may occur frequently versus infrequently in local environments, which should influence whether higher maximal growth rate is ‘affordable’ local adaptation favored by selection, despite pleiotropic consequences. On the other hand, total growth might be an adaptation less subject to antagonistic pleiotropy; e.g., evolution of larger body size (cells with decreased surface to volume ratio) may be less metabolically demanding than evolution of faster growth rate. These ideas warrant further investigation, because relative spatial and temporal changes in the selective factors presented by environments such as estuaries, household sink drains and the human body remain poorly understood. Our study highlights the potential for bacterial growth performance to be influenced by the habitat of origin, while also reminding that selection occurs at the phenotypic level and phenotypic traits may differ in their possibility for improving performance in the local habitat.

Our results are also consistent with other studies that warn researchers should be cautious about drawing conclusions over comparative growth performance, if their experiments look at only one growth parameter or challenge condition (Yang et al., 2008). For example, the estuary isolates showed a relatively higher maximal growth rate than other bacterial isolates in one natural medium (big bluestem leachate), but not in the minimal-glucose lab medium. Thus, the equivalent growth rate performance for these isolates in the defined lab medium is contrary to results observed in the undefined medium created from a natural environmental source. Although we included different challenge conditions, we note that all of our experiments were performed using 37°C incubation. Obviously, this temperature would seem more relevant for growth of *P. aeruginosa* isolates taken recently from the clinic, as opposed to directly from an estuary or household sink drain. By this logic, we might have observed superior growth of the clinical isolates across our entire study; because this was not seen, we cautiously dismiss incubation temperature as a potential bias in our study outcome. Still, it would be intriguing to test whether other incubation temperatures or media conditions (e.g., artificial human sputum) might alter the conclusions for maximal growth rate and total growth comparisons among the tested isolates. Last, we note that the method of isolation (nutrient rich versus nutrient poor medium) may bias which isolates are obtained from natural sources, in turn potentially affecting growth performance of test isolates and patterns of resource-use diversity across isolate collections in studies such as the current one (Aagot et al., 2001; see also Buck, 1974; Hattori, 1980; Hattori, 1981). However, we reject this possible confounding factor because all bacteria were isolated using *Pseudomonas* isolation agar, a nutrient rich agar medium.

## Conclusion

We observed that *P. aeruginosa* isolates that experienced different recent ecologies can grow robustly and similarly in artificial lab medium with glucose, and in two media derived from natural plant sources. However, depending on the challenge environment and growth parameter measured, subtle but statistically significant differences were sometimes observed; this indicates the usefulness of high-resolution, automated growth measurements for distinguishing differences in these bacteria. Given the importance of *P. aeruginosa* as an opportunistic human pathogen in some individuals (e.g., CF, severe burn, and immune-compromised patients), it bears emphasizing that very similar growth performance was observed across bacteria taken from seemingly different environments. These results underscore the phenotypic versatility of *P. aeruginosa* bacteria, an opportunistic pathogen found in both natural and human-built environments.

## Author Contributions

KD: main execution of the experiments, writing and editing of the manuscript. SR: statistical analyses, writing and editing of the manuscript. OO: experiments and data analysis, editing and commenting on the manuscript. MB: design of the experiments, editing and commenting on the manuscript. PR: design of the experiments, feedback on the manuscript. PT: design of the experiments, writing and editing of the manuscript.

## Conflict of Interest Statement

The authors declare that the research was conducted in the absence of any commercial or financial relationships that could be construed as a potential conflict of interest.

## Footnotes

1 http://www.evolvedmicrobe.com/CurveFitter/

2 http://www.evolvedmicrobe.com/CurveFitter/

## References

[B1] Aagot N. Nybroe O. Nielsen P. Johnsen K. (2001). An altered *Pseudomonas* diversity is recovered from soil by using nutrient-poor *Pseudomonas*-selective soil extract media. *Appl. Environ. Microbiol.* 67 5233–5239. 10.1128/AEM.67.11.5233-5239.2001 11679350 PMC93295

[B2] Bazire A. Diab F. Jebbar M. Haras D. (2007). Influence of high salinity on biofilm formation and benzoate assimilation by *Pseudomonas aeruginosa*. *J. Ind. Microbiol. Biotechnol.* 34 5–8. 10.1007/s10295-006-0087-2 16491361

[B3] Bozeman M. M. (2012). *Implications of the Quality, Quantity, and ‘Stickiness’ of Dissolved Organic Matter on Aquatic Ecosystem Function*. Ph.D. thesis, Yale University New Haven, CT 131.

[B4] Buchanan R. L. Whiting R. C. Damert W. C. (1997). When is simple good enough: a comparison of the Gompertz, Baranyi, and three-phase linear models for fitting bacterial growth curves. *Food Microbiol.* 14 313–326. 10.1006/fmic.1997.0125

[B5] Buck J. D. (1974). Effects of medium composition on the recovery of bacteria from sea water. *J. Exp. Mar. Biol. Ecol.* 15 25–34. 10.1016/0022-0981(74)90060-4 18515474

[B6] Burns J. L. Gibson R. L. McNamara S. Yim D. Emerson J. Rosenfeld M. (2001). Longitudinal assessment of *Pseudomonas aeruginosa* in young children with cystic fibrosis. *J. Infect. Dis.* 183 444–452. 10.1086/318075 11133376

[B7] Chan B. K. Sistrom M. Wertz J. E. Kortright K. E. Narayan D. Turner P. E. (2016). Phage selection restores antibiotic sensitivity in MDR *Pseudomonas aeruginosa*. *Sci. Rep.* 6:26717. 10.1038/srep26717 27225966 PMC4880932

[B8] Ciofu O. Mandsberg L. F. Bjarnsholt T. Wasserman T. Høiby N. (2010). Genetic adaptation of *Pseudomonas aeruginosa* during chronic lung infection of patients with cystic fibrosis: strong and weak mutators with heterogeneous genetic backgrounds emerge in *mucA* and/or *lasR* mutants. *Microbiology* 156 1108–1119. 10.1099/mic.0.033993-0 20019078

[B9] D’Argenio D. A. Wu M. Hoffman L. R. Kulasekara H. D. Déziel E. Smith E. E. (2007). Growth phenotypes of *Pseudomonas aeruginosa lasR* mutants adapted to the airways of cystic fibrosis patients. *Mol. Microbiol.* 64 512–533. 10.1111/j.1365-2958.2007.05678.x 17493132 PMC2742308

[B10] Delaney N. F. Kaczmarek M. E. Marx C. J. (submitted). Evaluating sources of bias when estimating microbial growth rates in microtiter plates and development of the open-source program Curve Fitter.

[B11] France M. T. Remold S. K. (2015). Interference competition among household strains of *Pseudomonas*. *Microb. Ecol.* 72 821–830. 10.1007/s00248-015-0652-1 26276409

[B12] Friman V.-P. Ghoul M. Molin S. Johansen H. K. Buckling A. (2013). *Pseudomonas aeruginosa* adaptation to lungs of cystic fibrosis patients leads to lowered resistance to phage and protist enemies. *PLoS One* 8:e75380. 10.1371/journal.pone.0075380 24069407 PMC3777905

[B13] Frimmersdorf E. Horatzek S. Pelnikevich A. Wiehlmann L. Schomburg D. (2010). How *Pseudomonas aeruginosa* adapts to various environments: a metabolomic approach. *Environ. Microbiol.* 12 1734–1747. 10.1111/j.1462-2920.2010.02253.x 20553553

[B14] Ghoul M. West S. A. Johansen H. K. Molin S. Harrison O. B. Maiden M. C. J. (2015). Bacteriocin-mediated competition in cystic fibrosis lung infections. *Proc. R. Soc. B* 282:20150972. 10.1098/rspb.2015.0972 26311664 PMC4571691

[B15] Hattori T. (1980). A note on the effect of different types of agar on plate count of oligotrophic bacteria in soil. *J. Gen. Appl. Microbiol.* 26 373–374. 10.2323/jgam.26.373

[B16] Hattori T. (1981). Enrichment of oligotrophic bacteria at microsites of soil. *J. Gen. Appl. Microbiol.* 27 43–55. 10.2323/jgam.27.43

[B17] Høiby N. (2006). *P. aeruginosa* in cystic fibrosis patients resists host defenses, antibiotics. *Microbe* 1 571–577. 10.1128/microbe.1.571.1

[B18] Holloway B. W. (1955). Genetic recombination in *Pseudomonas aeruginosa*. *J. Gen. Microbiol.* 13 572–581. 10.1099/00221287-13-3-572 13278508

[B19] Jelsbak L. Johansen H. K. Frost A. L. Thogersen R. Thomsen L. E. Ciofu O. (2007). Molecular epidemiology and dynamics of *Pseudomonas aeruginosa* populations in lungs of cystic fibrosis patients. *Infect. Immun.* 75 2214–2224. 10.1128/IAI.01282-06 17261614 PMC1865789

[B20] Jørgensen K. M. Wasserman T. Johansen H. K. Christiansen L. E. Molin S. Høiby N. (2015). Diversity of metabolic profiles of cystic fibrosis *Pseudomonas aeruginosa* during the early stages of lung infection. *Microbiology* 161 1447–1462. 10.1099/mic.0.000093 25873584

[B21] Khan N. H. Yoshikazu I. Kimata-Kino N. Esaki H. Nishino T. Nishimura M. (2007). Isolation of *Pseudomonas aeruginosa* from open ocean and comparison with freshwater, clinical and animal isolates. *Microb. Ecol.* 53 173–186. 10.1007/s00248-006-9059-3 17206394

[B22] Kidd T. J. Ritchie S. R. Ramsay K. A. Grimwood K. Bell S. C. Rainey P. B. (2012). *Pseudomonas aeruginosa* exhibits frequent recombination, but only a limited association between genotype and ecological setting. *PLoS One* 7:e44199. 10.1371/journal.pone.0044199 22970178 PMC3435406

[B23] Kilham S. S. Kreeger D. A. Lynn S. G. Goulden C. E. Herrera L. (1998). COMBO: a defined freshwater culture medium for algae and zooplankton. *Hydrobiologia* 377 147–159. 10.1023/A:1003231628456

[B24] Kimata N. Nishino T. Suzuki S. Kogure K. (2003). *Pseudomonas aeruginosa* isolated from marine environments. *Microb. Ecol.* 47 41–47. 10.1007/s00248-003-1032-9 15259268

[B25] Kirov S. M. Webb J. S. O’May C. Y. Reid D. W. Woo J. K. K. Rice S. A. (2007). Biofilm differentiation and dispersal in mucoid *Pseudomonas aeruginosa* isolated from patients with cystic fibrosis. *Microbiology* 153 3264–3274. 10.1099/mic.0.2007/009092-0 17906126

[B26] Klockgether J. Munder A. Neugebauer J. Davenport C. F. Stanke F. Larbig K. D. (2010). Genome diversity of *Pseudomonas aeruginosa* PA01 laboratory strains. *J. Bacteriol.* 192 1113–1121. 10.1128/JB.01515-09 20023018 PMC2812968

[B27] Kokare C. R. Chakraborty S. Khopade A. N. Mahadik K. R. (2009). Biofilm: importance and applications. *Ind. J. Biotechnol.* 8 159–168.

[B28] Mahenthiralingam E. Campbell M. E. Speert D. P. (1994). Nonmotility and phagocytic resistance of *Pseudomonas aeruginosa* isolates from chronically colonized patients with cystic fibrosis. *Infect. Immun.* 62 596–605. 8300217 10.1128/iai.62.2.596-605.1994 PMC186146

[B29] Mesaros N. Nordmann P. Plésiat P. Roussel-Delvallez M. Van Eldere J. Glupczynski Y. (2007). *Pseudomonas aeruginosa*: resistance and therapeutic options at the turn of the new millennium. *Clin. Microbiol. Infect.* 13 560–578. 10.1111/j.1469-0691.2007.01681.x 17266725

[B30] Mueller L. D. Guo P. Z. Ayala F. J. (1991). Density-dependent natural selection and trade-offs in life history traits. *Science* 253 433–435. 10.1126/science.1907401 1907401

[B31] Murray T. S. Ledizet M. Kazmierczak B. I. (2010). Swarming motility, secretion of type 3 effectors and biofilm formation phenotypes exhibited within a large cohort of *Pseudomonas aeruginosa* clinical isolates. *J. Med. Microbiol.* 59 511–520. 10.1099/jmm.0.017715-0 20093376 PMC2855384

[B32] Novak M. Pfeiffer T. Lenski R. E. Sauer U. Bonhoeffer S. (2006). Experimental tests for an evolutionary trade-off between growth rate and yield in *E. coli*. *Am. Nat.* 168 242–251. 10.2307/3844729 16874633

[B33] Palmer G. C. Palmer K. L. Jorth P. A. Whiteley M. (2010). Characterization of the *Pseudomonas aeruginosa* transcriptional response to phenylalanine and tyrosine. *J. Bacteriol.* 192 2722–2728. 10.1128/JB.00112-10 20304990 PMC2876504

[B34] Purdy-Gibson M. E. France M. Hundley T. C. Eid N. Remold S. K. (2015). *Pseudomonas aeruginosa* in CF and non-CF homes is found predominantly in drains. *J. Cyst. Fibros.* 14 341–346. 10.1016/j.jcf.2014.10.008 25443472

[B35] Qualls R. G. (2000). Comparison of the behavior of soluble organic and inorganic nutrients in forest soils. *For. Ecol. Manage* 138 29–50. 10.1016/S0378-1127(00)00410-2

[B36] Rau M. H. Marvig R. L. Ehrlich G. D. Molin S. Jelsbak L. (2012). Deletion and acquisition of genomic content during early stage adaptation of *Pseudomonas aeruginosa* to a human host environment. *Environ. Microbiol.* 14 2200–2211. 10.1111/j.1462-2920.2012.02795.x 22672046

[B37] Raymond P. A. Hopkinson C. S. (2003). Ecosystem modulation of dissolved carbon age in a temperate marsh-dominated estuary. *Ecosystems* 6 694–705. 10.1007/s10021-002-0213-6

[B38] Remold S. K. Brown C. K. Farris J. E. Hundley T. C. Perpich J. A. Purdy M. E. (2011). Differential habitat use and partitioning by *Pseudomonas* species in human homes. *Microb. Ecol.* 62 505–517. 10.1007/s00248-011-9844-5 21503776

[B39] Rodríguez-Rojas A. Oliver A. Blázquez J. (2012). Intrinsic and environmental mutagenesis drive diversification and persistence of *Pseudomonas aeruginosa* in chronic lung infections. *J. Infect. Dis.* 205 121–127. 10.1093/infdis/jir690 22080096

[B40] Ruimy R. Genauzeau E. Barnabe C. Beaulieu A. Tibayrenc M. Andremont A. (2001). Genetic diversity of *Pseudomonas aeruginosa* strains isolated from ventilated patients with nosocomial pneumonia, cancer patients with bacteremia, and environmental water. *Infect. Immun.* 69 584–588. 10.1128/IAI.69.1.584-588.2001 11119558 PMC97924

[B41] SAS Institute Inc. (2011). *9.3 User’s Guide*. Cary, NC: SAS Institute, Inc.

[B42] Schechner V. Nobre V. Kaye K. S. Leshno M. Giladi M. Rohner P. (2009). Gram-negative bacteremia upon hospital admission: When should *Pseudomonas aeruginosa* be suspected? *Clin. Infect. Dis.* 48 580–586. 10.1086/596709 19191643

[B43] Schmidt K. D. Tümmler B. Römling U. (1996). Comparative genome mapping of *Pseudomonas aeruginosa* PAO with *P. aeruginosa* C, which belongs to a major clone in cystic fibrosis patients and aquatic habitats. *J. Bacteriol.* 178 85–93. 10.1128/jb.178.1.85-93.1996 8550447 PMC177624

[B44] Selezska K. Kazmierczak M. Müsken M. Garbe J. Schobert M. Häussler S. (2012). *Pseudomonas aeruginosa* population structure revisited under environmental focus: impact of water quality and phage pressure. *Environ. Microbiol.* 14 1952–1967. 10.1111/j.1462-2920.2012.02719.x 22390474

[B45] Workentine M. Surette M. G. (2011). Complex *Pseudomonas* population structure in cystic fibrosis airway infections. *Am. J. Respir. Crit. Care Med.* 183 1581–1583. 10.1164/rccm.201105-0776ED 21693712

[B46] Yang L. Haagensen J. A. Jelsbak L. Johansen H. K. Sternberg C. Høiby N. (2008). In situ growth rates and biofilm development in chronic lung infections. *J. Bacteriol.* 190 2767–2776. 10.1128/JB.01581-07 18156255 PMC2293235

[B47] Yoshpe-Purer Y. Golderman S. (1987). Occurrence of *Staphylococcus aureus* and *Pseudomonas aeruginosa* in Israeli coastal water. *Appl. Environ. Microbiol.* 53 1138–1141. 3111367 10.1128/aem.53.5.1138-1141.1987 PMC203821

