# Supplementary material for: Generalized Growth of Estuarine, Household and Clinical Isolates of Pseudomonas aeruginosa
Source: Front Microbiol. 2018 Feb 27;9:305. doi: 10.3389/fmicb.2018.00305 (PMC5863524; doi:10.3389/fmicb.2018.00305)
Supplement: Supplementary file 1 [file Image_1.PDF]

*Supplementary Material*

# Generalized growth of estuarine, household and clinical isolates of *Pseudomonas aeruginosa*

**Kelly E. Diaz, Susanna K. Remold, Ogochukwu Onyiri, Maura Bozeman, Peter Raymond, and Paul E. Turner***

*** Correspondence:** Corresponding Author: paul.turner@yale.edu

## 1.1 Supplementary Figures

**Supplementary Figure 1.** This scatterplot depicts mean data for growth rate and total growth estimated for each isolate in the current study, with the assay Media indicated by label (BS - big bluestem grass, SM - sugar maple leaves, DM - DM10 lab medium), and the Habitat of Origin by color. The results presented in Fig. 1 and Table 2 are clearly seen in Fig. S1 as well. First, there is a stronger separation by Media than by Habitat of Origin both vertically (total growth) and horizontally (growth rate). Second, there is some clustering of isolates from the same habitat of origin within the larger clusters of assay media (indicating a Media*Origin interaction effect), also seen along both axes.

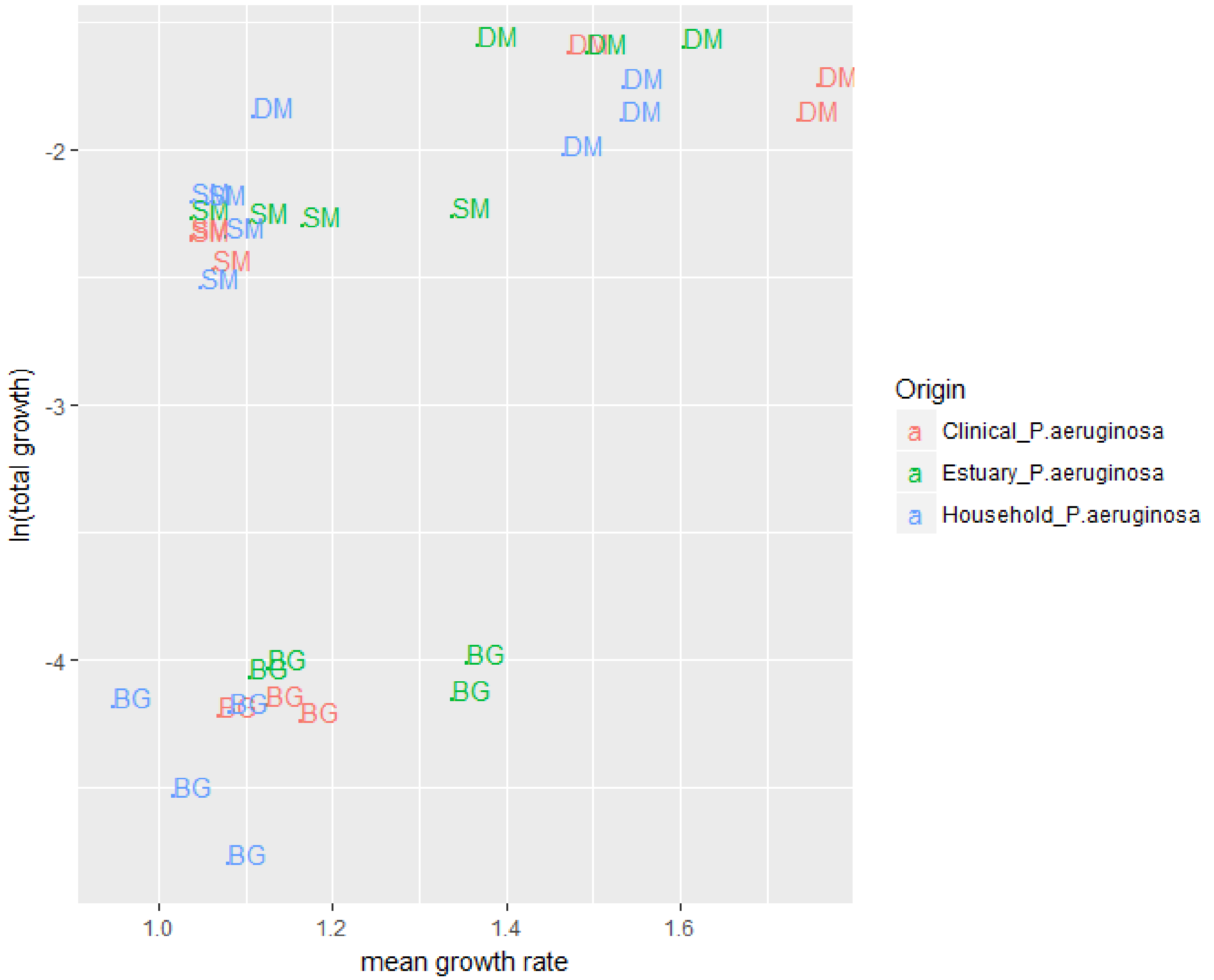

DM
SM
BG
ln(total growth)
-2
-3
-4
1.0
1.2
1.4
1.6
mean growth rate
Origin
Clinical_P.aeruginosa
Estuary_P.aeruginosa
Household_P.aeruginosa
